# Valorizing Brazilian Propolis Residue: Comprehensive Characterization for Sustainable Reutilization Strategies

**DOI:** 10.3390/plants14131989

**Published:** 2025-06-29

**Authors:** Agnese Santanatoglia, Laura Acquaticci, Maria Cristina Marcucci, Filippo Maggi, Carlos Rocha Oliveira, Giovanni Caprioli

**Affiliations:** 1 School of Pharmacy, Chemistry Interdisciplinary Project (ChIP), University of Camerino, Via Madonna delle Carceri 9/B, 62032 Camerino, Italy; agnese.santanatoglia@unicam.it (A.S.); laura.acquaticci@unicam.it (L.A.); filippo.maggi@unicam.it (F.M.); 2 Research and Innovation Coffee Hub, Via Emilio Betti 1, 62020 Belforte del Chienti, Italy; 3 Department of Biosciences and Oral Diagnosis, ICT-Unesp, Sao Jose dos Campos 12245-000, Brazil; cristina.marcucci@unesp.br; 4 Gap Laboratory of Biotechnology, Sao Jose dos Campos 12243-020, Brazil; carlos.rocha@unifesp.br; 5 Post-Graduate Program in Biomedical Engineering, Federal University of Sao Paulo, Sao Jose dos Campos 12331-280, Brazil

**Keywords:** propolis residue, bioactive compounds, volatile compounds, circular economy, nutritional profile, by-products, antioxidant activity

## Abstract

This study presents the first comprehensive characterization of Brazilian propolis residue, revealing its rich content of bioactive compounds, essential nutrients, and volatile substances, showcasing its potential for sustainable utilization. The term “residue” refers to the solid by-product remaining after ethanolic extraction of raw propolis, which is typically discarded, despite retaining significant nutritional value. HPLC-ESI-MS/MS analysis identified significant concentrations of *p*-coumaric acid (637.80 mg/kg), chlorogenic acid (497.93 mg/kg), kaempferol (295.82 mg/kg), and caffeic acid (115.11 mg/kg); while HPLC-DAD revealed also artepillin-C (56.56 mg/kg), illustrating strong antioxidant properties. Nutritional analyses showed high moisture content (37.08%), protein (12.56%) and dietary fiber (24.2%). Additionally, the mineral profile highlighted potassium (9800 mg/kg), phosphorus (2520 mg/kg), and calcium (2100 mg/kg). Volatile compounds analysis via HS-SPME-GC-MS identified a diverse class of components, predominantly terpenoids such as α-pinene (20.09%) and caryophyllene (9.76%), suggesting potential applications in fragrance and flavor industries. The multifunctional nature of propolis residue aligns with circular economy principles and highlights its value as a resource for diverse applications.

## 1. Introduction

The utilization of biomass residues represents a critical frontier in the advancement of sustainable technologies and the circular economy. To safeguard their hive, the *Apis mellifera* L. bees synthesize a resinous substance known as propolis, which primarily consists of resin (60%), and contains smaller proportions of wax, essential oils, and various other components [1,2]. The valorization of propolis has gained increasing attention due to its diverse biological activities, including antioxidant, antibacterial, antiviral, and anti-inflammatory properties, which are attributed to its rich chemical composition of phenolic acids, flavonoids, terpenes, and sugars [3,4,5,6]. These components have been identified as key contributors to propolis’ health-promoting properties, with potential therapeutic roles in wound healing, treatment of ulcers, sore throats, infections, and chronic diseases such as diabetes, hypertension, and cardiovascular diseases [3]. The versatile use of propolis in medical and dental fields, alongside its different pharmaceutical forms (capsules, tablets, tinctures, creams, and mouthwashes), emphasizes its potential industrial application [7,8,9]. Emerging research also highlights propolis’ capacity as a natural food preservative by enhancing shelf-life, quality, and stability of food products [10,11,12,13]. The use of propolis extracts and specialized coatings has been shown to significantly enhance the freshness of a wide range of fruits and vegetables. This suggests that propolis could also be beneficial in cosmetic products, indicating its potential for wider applications beyond food preservation [12]. Recognized for its safety (GRAS status), propolis has been considered to reduce microbial contamination and improve food quality during storage [10,13,14], although such designation depends on the specific botanical source, chemical profile, and intended use of the extract. Despite the extensive literature on raw propolis, limited attention has been paid to the industrial residue generated after extraction. Globally, the propolis industry produces an estimated 2000–3000 tons annually, with Brazil contributing over 30% of the total volume, primarily through the production of green propolis [12]. The ethanolic extraction process generates solid residues accounting for approximately 25–35% of the initial raw material weight.

The characterization of propolis residues, influenced by botanical origin, local flora and production season [15,16], reveals a complex chemical profile with over 300 substances. The observed variety underscores how the effectiveness of propolis in fighting microbes and protecting against oxidation can vary. This is directly related to the different types of phenolic compounds found in propolis, which differ in structure and concentration, depending on the region. This means that the antimicrobial and antioxidant properties of propolis can change, depending on place of origin, due to variations in its chemical composition [11,17,18]. Moreover, studying the impact of different extraction processes on the mineral content of propolis tinctures represents an intriguing field, for nutraceutical applications [19]. However, the environmental impact of propolis residue disposal, especially in large-scale production, necessitates a closer examination. Despite its unique smell, research suggests that using specific amounts of propolis powder in industrial food production can effectively control its impact on the flavor of food products [20]. This work aims to find new potential uses of propolis residues by evaluating the nutritional profile (moisture, protein, total fats, dietary fiber, ash, carbohydrates, sugar composition, key elements, and minerals), the polyphenols content (using HPLC-MS/MS), the antioxidant properties (using UV-Vis spectrophotometric assays), and the volatile profile (using GC-MS). Recognizing the challenges in valorizing propolis residues, this study aims to find innovative solutions aligned with industrial applications, environmental sustainability, and circular economy principles.

## 2. Materials and Methods

### 2.1. Reagents and Standards

Cyanidin-3-glucoside chloride, delphinidin-3,5-diglucoside chloride, delphinidin-3-galactoside chloride, petunidin-3-glucoside chloride, malvidin-3-galactoside chloride, quercetin-3-glucoside, and kaempferol-3-glucoside were purchased from PhytoLab (Vestenbergsgreuth, Germany). The remaining 31 analytical standards of the 38 phenolic compounds were supplied by Sigma-Aldrich (Milan, Italy). Individual stock solutions of each analyte, at a concentration of 1000 mg L^−1^, were prepared by dissolving pure standards in HPLC-grade methanol and storing them in glass stoppered bottles at 4 °C, except for anthocyanins, which were stored at −15 °C until analysis. Standard working solutions at various concentrations were prepared daily by appropriate dilution of the stock solutions with HPLC-grade methanol. HPLC-grade methanol was supplied by Sigma-Aldrich (Milano, Italy). Deionized water (>18 MΩ cm resistivity) was further purified using a Milli-Q SP Reagent Water System (Millipore, Bedford, MA, USA). All solvents and solutions were filtered through a 0.2 μm polyamide filter from Sartorius Stedim (Goettingen, Germany). All chemicals and reagents were analytical grade. Before HPLC analysis, all samples were filtered with a Phenex™ RC 4 mm 0.2 μm syringeless filter, Phenomenex (Castel Maggiore, BO, Italy). The alkane mixture (C_7_–C_30_) was purchased from Sigma-Aldrich (Milano, Italy). The Folin–Ciocolteu reagent, sodium carbonate (Na_2_CO_3_), gallic acid (C_7_H_6_O_5_), ferric chloride hexahydrate (FeCl_3_·6H_2_O), and Trolox (6-hydroxy-2,5,7,8-tetramethylchroman-2-carboxylic acid) were purchased from Sigma-Aldrich (St. Louis, MO, USA). The DPPH (2,2-diphenyl-1-picrylhydrazyl radical) was obtained from Glentham Life Sciences (Corsham, UK).

### 2.2. Crude Propolis Samples and Industrial Residues

The raw organic propolis and its associated industrial residues, after the ethanolic extraction process, were supplied by Green Eucalypt Propolis Ltd, located in Pindamonhangaba, State of Sao Paulo, Brazil (coordinates: Latitude: 22°55′25″ S, Longitude: 45°27′35″ W) [21]. The crude propolis was supplied in its natural, piece-form state. Crude propolis was industrially extracted using 70% ethanol. At the end of the process, it was filtered, the wax was separated by filtration, and the dry insoluble residue was called “propolis residue”. This industrial waste was supplied in a dehydrated state, as dry residue, after ethanolic extraction. To prepare the wet propolis residue for subsequent analysis, a two-step drying process was employed. Initially, the residue was allowed to air dry at room temperature to reduce the ethanol content through natural evaporation. Subsequently, to ensure complete removal of the solvent, the material was placed in an oven and dried at a controlled temperature of 60 °C (Figure S1). Brazilian propolis residue provided by Apis Flora was classified as *brown propolis*, primarily obtained from a mixture of plant sources including *Baccharis dracunculifolia* and *Dalbergia ecastaphyllum*. The residue was derived from an industrial-scale hydroalcoholic extraction (ethanol:water, 70:30) performed by Apis Flora Ltd.a., Brazil, to produce standardized propolis extract (EPP-AF^®^). The remaining solid fraction was then dried and stored before analysis.

### 2.3. Preparation of Extracts

For the extraction, 1 g of propolis was extracted using 20 mL of an ethanol/water mixture (70/30 *v*/*v*), maintaining a matrix/solvent ratio of 1:20. The extraction was achieved through sonication for 40 min at 25 °C and a frequency of 59 Hz, with manual stirring occurring every 10 min to keep the powder in contact with the solvent throughout the process. Subsequently, the sample was subjected to vortex and centrifuge, the supernatant was collected, and the residue was washed with 5 mL of ethanol. The extract was then evaporated, dissolved in 10 mL of methanol, and freeze-dried. Then, 1 mL of this solution was placed into a centrifuge tube and re-centrifuged to remove any precipitates, filtered, and injected into HPLC for analysis. Meanwhile, for HPLC-DAD analysis, a solution with 0.15 g of propolis and 5 mL of methanol, HPLC grade, was prepared. The extracts were performed in triplicate (*n* = 3) to ensure reproducibility.

### 2.4. HPLC-ESI-MS/MS Analysis

The analysis of 36 bioactive compounds was conducted following a method previously described [22,23,24], with some modifications. Briefly, 2 μL of filtered sample was injected in an Agilent 1290 Infinity series HPLC system linked to an Agilent Triple Quadrupole 6420 from Agilent Technology (Santa Clara, CA, USA), equipped with an electrospray ionization (ESI) source, able to operate in both negative and positive ionization modes. Data collection was performed using the MassHunter MS Optimizer software (Agilent). Chromatographic separation was achieved with a Synergi Polar–RP C_18_ column (250 mm × 4.6 mm, 4 μm) provided by Phenomenex (Cheshire, UK), employing a mobile phase composed of (A) water and (B) methanol, each containing 0.1% formic acid, at a flow rate of 0.8 mL/min. The gradient elution schedule was developed as follows: 0–1 min, isocratic phase at 20% B; 1–25 min, linear increase from 20% to 85% B; 25–26 min, isocratic phase at 85% B; 26–32 min, return to 20% B, remaining for 8 min. The ESI source’s drying gas was heated to 350 °C, with a nebulizer pressure at 55 psi, a capillary voltage of 4000 V and a gas flow rate maintained at 12,000 mL/min. Quantification was based on the integrated peak areas of the predominant product ions after detection through dynamic–multiple reaction monitoring mode. Specific ion transitions and mass spectrometry parameters for each compound are detailed in Table S1.

### 2.5. HPLC-DAD Analysis

The quantification of Artepillin-C (3,5-diprenyl-4-hydroxycinnamic acid) was performed using a high-performance liquid chromatography (HPLC) system (Agilent 1100, Model G1311A, Santa Clara, CA, USA) equipped with a quaternary pump and auto-sampler. A Nucleosil C_18_ column (150 × 4.6 mm, 5 μm) (Supelco, Lot: 608010560) was used for separation. The mobile phase consisted of (A) ultrapure water with 5% formic acid (Merck, 99%) and (B) 100% methanol, with a flow rate set at 1 mL/min and a linear gradient. The total analysis time was set at 50 min, with detection at 280 and 320 nm using a diode array detector (DAD). Artepillin-C was quantified by comparison with an authentic standard, following a calibration curve.

### 2.6. Spectrophotometric Assays

#### 2.6.1. Antioxidant Activity (DPPH)

Antioxidant activity was determined using the DPPH method, spectrophotometrically evaluating the decay of the 2,2-diphenyl-1-picril-hidrazyl radical by antioxidant substances. These tests were carried out on the matrix after dilution 1:100 in water (100 mg in 10 mL), following the procedure described by [25], with some modifications. Briefly, 0.5 mL of the diluted propolis was mixed with 4.5 mL of an ethanolic DPPH solution (0.1 mM). After 30 min of incubation in the dark at room temperature, the decrease in the DPPH radical was measured spectrophotometrically at 517 nm using an Agilent Cary 8454 UV-Vis spectrophotometer. Trolox was used as the reference antioxidant, and the results were expressed as mg Trolox equivalent (TE)/kg of extract.

#### 2.6.2. Determination of Total Phenolic Content (TPC) and Total Flavonoid Content (TFC)

TPC was determined spectrophotometrically according to [25], with some modifications. All samples were diluted 1:100 (100 mg in 10 mL) in water. Briefly, 0.5 mL of the sample solution was added to the tubes, then 2.5 mL of the Folin–Denis’s reagent solution and 7 mL of Na_2_CO_3_ solution (7.5% *w*/*w* in water) were added. The reaction mixture was allowed to stand in the dark at room temperature for 2 h, and the absorbance was measured at 765 nm. Quantification of TPC in the extracts was performed using a gallic acid calibration curve and was expressed as mg gallic acid equivalent (GAE)/kg of extract.

The TFC of the different extracts was determined according to a method described by [25], with slight variations. All samples were diluted 1:100 (100 mg in 10 mL) in water. In a 15 mL tube, 0.5 mL of the sample solution, 0.15 mL NaNO_2_ (0.5 M), 3.2 mL methanol (30% *v*/*v*), and 0.15 mL AlCl_3_·6H_2_O (0.3 M) were mixed. After 5 min, 1 mL of NaOH (1 M) was added. The solution was mixed, and the absorbance was measured at 506 nm compared to the blank. The standard calibration curve for TFC was prepared using a rutin standard solution, according to the same procedure as described above. TFC was expressed as mg rutin equivalent (RE)/kg of extract.

### 2.7. GC-MS Analysis

The extraction was performed using an HS-SPME method and the analysis was conducted on a GC-MS instrument. Briefly, 3 g of sample was put in a 20 mL vial, and it was tightly clapped with a PTFE/silicon septum. Samples were incubated at 60 °C for 15 min under stirring (250 rpm with 5 s of on-time and 2 s of off-time) and then extracted for 30 min with a Divinylbenzene/Carbon-Wide Range/Polydimethylsiloxane DVB/C-WR/PDMS (80 µm) fiber. The fiber was conditioned for 10 min at 250 °C and then inserted inside the headspace of the sample vial with a speed of 20 mm·s^−1^ and a penetration depth of 35 mm. The extraction was performed and then the fiber was inserted into the injector port at a speed of 100 mm·s^−1^ and a penetration depth of 40 mm. The desorption occurred at 250 °C for 2 min. After desorption, the fiber was conditioned at 250 °C for 5 min.

The GC-MS system was composed of an Agilent 8890 GC coupled to an Agilent 5977B MSD quadrupole detector with an electron ionization (EI) source (Santa Clara, CA, USA). The system was equipped with an autosampler PAL RTC 120 System. The injector temperature was set at 250 °C and the liner used was recommended for SPME injection, namely, Inlet liner, Ultra Inert, splitless, straight, 0.75 mm id from Agilent. The gas carrier was helium at a flow rate of 1 mL·min^−1^. A DB-WAX UI capillary column (60 m × 0.25 mm × 0.25 μm) was used. Thermal desorption was carried out at 250 °C in a splitless mode for 2 min. Oven temperature was set at 35 °C held for 3 min, increased up to 200 °C at 5 °C/min, increased up to 250 °C at 15 °C/min, held for 5 min. The temperatures of the ionization source and the mass analyzer were set at 230 and 150 °C, respectively. The acquisition was carried out in SCAN mode (35–450 *m*/*z*).

Volatile compounds were identified through the comparison of their mass spectra with those of NIST library (US National Institute of Standards and Technology) in combination with the calculation of their experimental linear retention indexes, which have been compared to those reported in the literature. Compound abundances were determined using the relative percentage of the area (%) of each peak that was calculated by dividing the area of each component by the total area of all separated components. Data results were managed using MSD ChemStation Software (Agilent, Version G1701DA D.01.00, Santa Clara, CA, USA) [26].

### 2.8. Nutrients

Samples were analyzed to determine their nutrient composition through AOAC procedures [27]. Moisture content was determined by oven drying (24 h, 133 °C) until a constant weight. Ash content was determined by incineration at 600 ± 15 °C; fat level was obtained by Soxhlet extraction of a fixed quantity of samples with petroleum ether; while the Kjeldahl method was used for the protein content; after acidic hydrolysis, dietary fiber levels were determined through a gravimetric method and total carbohydrates were determined by difference. Total energy was calculated according to the following equations: *Energy (kcal) = 4 X (g protein + g carbohydrate) + 9 X (g lipid)*. Sugars were determined using high-performance anion exchange chromatography with a pulsed amperometric detection method (HPAEC-PAD) [28]. The mineral composition was performed by the digestion of dried samples with microwaves, then analysis was performed using inductively coupled plasma mass spectrometry (ICP-MS) [29].

### 2.9. Statistical Analysis

All experimental data reported in the tables were subject to one-way and two-way analysis of variance (ANOVA) and are reported as triplicate average values and standard deviations. Whenever a significant difference was observed (*p* < 0.05), a post hoc multiple comparison test using Fisher’s LSD test was performed (α = 0.05), while the statistical analysis reported for the figures was processed using the XLSTAT 2018.2 software (AddinSoft, Paris, France).

## 3. Results and Discussion

### 3.1. Bioactive Compound Analysis in Propolis Residue

**Figure 1:**
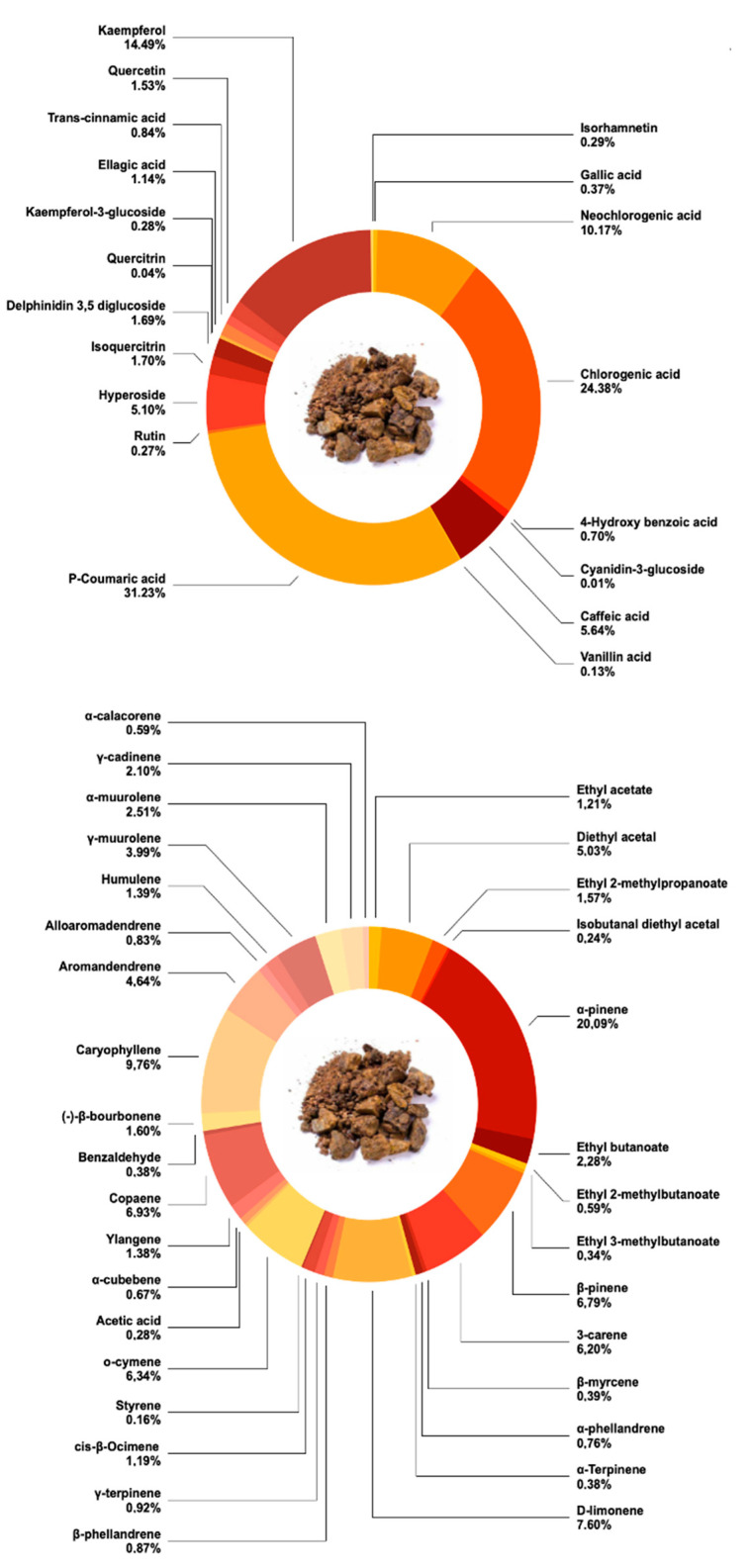
Bioactive and volatile compounds in Brazilian propolis residue. This figure illustrates the predominant phenolic acids, such as *p*-coumaric acid (637.80 ± 27.35 mg/kg), chlorogenic acid (497.93 ± 28.33 mg/kg), and kaempferol (295.82 ± 11.67 mg/kg), along with significant concentrations of volatile compounds like α-pinene (20.09%) and caryophyllene (9.76%), in propolis residue. These components highlight the residue’s potential for antioxidant activity and applications in fragrance and flavor industries.

The analysis of propolis residue, derived from raw propolis processing, reveals a rich composition of bioactive phenolic compounds, crucial for valorizing this biomass in sustainable applications. Phenolic compounds contribute to sustainability being derived from natural sources, offering a natural alternative to synthetic antioxidants in various applications [30], reducing reliance on chemical processes and enhancing biodegradability [23,24,31]. Utilizing HPLC-ESI-MS/MS, a diverse array of phenolic compounds was quantified (Table 1) (Figure 1), underscoring the potential of propolis residue as a source of natural antioxidants.

The study identified significant quantities of *p*-coumaric acid (637.80 ± 27.35 mg/kg), chlorogenic acid (497.93 ± 28.33 mg/kg), and kaempferol (295.82 ± 11.67 mg/kg) as the predominant compounds, alongside notable concentrations of neochlorogenic acid (207.67 ± 19.32 mg/kg). Several studies reported that propolis contains bioactive compounds such as phenolic compounds, flavonoids, and antioxidant compounds [32,33,34]. Moreover, caffeic acid (115.11 ± 6.51 mg/kg) was also detected in considerable amounts, further emphasizing the complexity and richness of propolis residue in bioactive compounds. The total quantified bioactive compounds by HPLC-ESI-MS/MS amounted to 2041.34 mg/kg dry weight, showcasing the substantial phenolic content present in propolis residue.

Also, Artepillin-C, a prenylated phenolic compound primarily found in Brazilian green propolis, known for its potent antioxidant, anti-inflammatory, and anticancer properties, contributing to the overall bioactivity of propolis, was identified by HPLC-DAD (56.56 ± 1.75 mg/kg) in a good concentration.

These findings are consistent with those of [35,36] who noted the antimicrobial and anti-inflammatory properties of propolis. Similarly, [37], emphasized its health-promoting applications. And according also, with the work of [38], who confirmed the superior antioxidant efficacy of propolis residues compared to other natural sources, including fruits and vegetables known for their health-promoting properties. For instance, volatile oil extracted from Brazilian brown propolis showed a DPPH IC_50_ of 25.0 µg/mL, which is considerably lower than the IC_50_ values typically reported for fruit-derived by-products, such as strawberry extracts (IC_50_ ≈ 150–230 µg/mL) and apple pomace (IC_50_ ≈ 200–400 µg/mL), supporting the enhanced antioxidant potential of propolis residue [39]. Based on the studies by [40], the concentration of *p*-coumaric acid in raw propolis, prior to extraction, is significantly higher than the values reported in other studies. For instance, [41] identified concentrations were greater than 300 mg/kg. Our findings of 637.80 ± 27.35 mg/kg for *p*-coumaric acid in propolis residues align more closely with these studies, supporting the idea that propolis is richer in this compound. In contrast, [42] found European propolis to have a lower kaempferol concentration up to 50 mg/kg. In comparison, this study identified a higher kaempferol content (295.82 ± 11.67 mg/kg), suggesting greater antioxidant potential in the examined propolis residues.

Moreover, comparing these results with berries such as blueberries, raspberries, and strawberries [43], in which the total content of phenolic compounds ranges from 1500 to 5000 mg/kg fresh weight, propolis residues were shown to be richer in these compounds, with a total phenolic content of 2041.34 mg/kg dry weight, underscoring propolis residue’s richness as a natural antioxidant source. Then, vegetables like spinach, cabbage, and broccoli are known for their phenolic content, with values varying significantly from 1000 to 2500 mg/kg fresh weight [44]. These analyses underscore the superior antioxidant potential of propolis residue, exceeding levels found in some natural sources and highlighting its value for sustainable applications.

### 3.2. Spectrophotometric Assays in Propolis Residue

#### 3.2.1. Total Phenolic Content (TPC) and Total Flavonoid Content (TFC)

Spectrophotometric assays have uncovered a high total phenolic content (TPC) of 16,726.67 ± 36.07 mg GAE/kg DW and a total flavonoid content (TFC) of 6802.96 ± 32.40 mg RE/kg DW in propolis residue (Table 2); however, TPC and TFC may be affected by matrix interference such as waxes and residual sugars, particularly in these samples.

According to [32], the chemical composition of propolis residue is like that of raw propolis, considering total phenolic content (TPC) and total flavonoid content (TFC). These findings are lower than those previously reported for this matrix but still surpass the phenolic and flavonoid values observed in various agricultural and industrial by-products.

For example, our TPC value remains higher, compared to 8000–15,000 mg GAE/kg DW range found in grape seeds by [45] and the 5000–22,000 mg GAE/kg DW in grape pomace reported by [46]. Similarly, the TFC values in our study also exceed the 6000–12,000 mg RE/kg DW reported for Italian grape pomaces by [47], highlighting the unique bioactive potential of propolis residue. Comparatively, [1] analyzed various samples of raw propolis, revealing a TPC range of approximately 20,000–30,000 mg GAE/kg DW, higher than these findings. Also, compared to the values reported by [48], which indicate a TPC range of 8000–15,000 mg GAE/kg DW for various agricultural by-products, these propolis residue samples demonstrated a higher total phenolic content (16,726.67 ± 36.07 mg GAE/kg DW). This underscores the superior bioactive potential of propolis residue, highlighting its promise as a valuable source of natural antioxidants.

#### 3.2.2. Antioxidant Activity (DPPH Assay)

The DPPH assay results confirmed the significant antioxidant capacity of propolis residue, with a value of 2921.61 mg TE/kg DW; it is important to recognize that in vitro assays like DPPH may overestimate antioxidant capacity due to the contribution of compounds that are not necessarily bioavailable in vivo. This activity aligns with the high concentrations of phenolics and flavonoids, demonstrating propolis residue’s efficacy in neutralizing free radicals and mitigating oxidative stress. Our findings coincide those of [49], who observed a direct correlation between phenolic content and antioxidant activity in winemaking by-products, although they reported a lower antioxidant activity (500–1500 mg TE/kg DW) than those observed in propolis residue.

### 3.3. Analysis of Volatile Compounds

The chemical composition of volatile compounds in propolis residue, performed by HS-SPME-GC-MS, is reported in Table 3 and Figure 1.

A total of 33 compounds have been identified, mainly terpenoids (68.41%), hydrocarbons (13.42%), acetals (5.27%), esters (4.78%), flavonoids (4.64%), alkaloids (1.60%), aldehydes (0.38%), and organic acids (0.28%). Among the terpenoids, monoterpenes turn out to be the predominant ones (42.63%), mainly represented by α-pinene (20.09%), followed by the sesquiterpenes (21.83%), mainly represented by caryophyllene (9.76%). Terpenes, including sesquiterpenes and monoterpene hydrocarbons, are also identified as some of the major volatile compounds in propolis [50,51,52]. These two compounds are the most abundant in the propolis residue, followed by limonene (7.60%), copaene (6.93%), β-pinene (6.79%), o-cymene (6.34%), and 3-carene (6.20%). Many of these compounds, particularly monoterpenes such as α-pinene, limonene, and β-pinene, are widely recognized for their aromatic, antimicrobial, and antioxidant properties. α-Pinene, for example, has been used in the fragrance industry due to its pine-like scent, while limonene is employed as a flavoring agent in food and beverages and as a natural solvent in green cleaning products [53]. Additionally, caryophyllene and copaene exhibit anti-inflammatory and antifungal activity, making them valuable for pharmaceutical and cosmetic applications [54]. These properties highlight the potential of propolis residue volatiles for use in natural flavoring, fragrance formulations, and functional product development. These data agree with those reported in the literature; in fact, studies on the volatile profile of propolis reported that α-pinene is present at a percentage between 1.2 and 46.5%, caryophyllene 6.2–21.7%, limonene 11.6%, copaene 8.2–22.19%, and β-pinene 2.0–21.8% [55,56,57,58]. The wide range of percentages found in the literature on propolis may be related to different geographical origins and biomes [1,59], isolation, and analytical techniques [60]. Propolis volatiles may be used as markers in the identification of the propolis botanical origin and its characterization [11]. Anyway, Ikeda et al. found that the most abundant compound in the essential oil of dried residue of propolis was α-pinene (16.25%), followed by ethyl benzoate (7.80%) and β-pinene (5.15%) [2].

### 3.4. Nutrient Composition of Propolis Residue

#### 3.4.1. Macronutrient Profile

**Figure 2:**
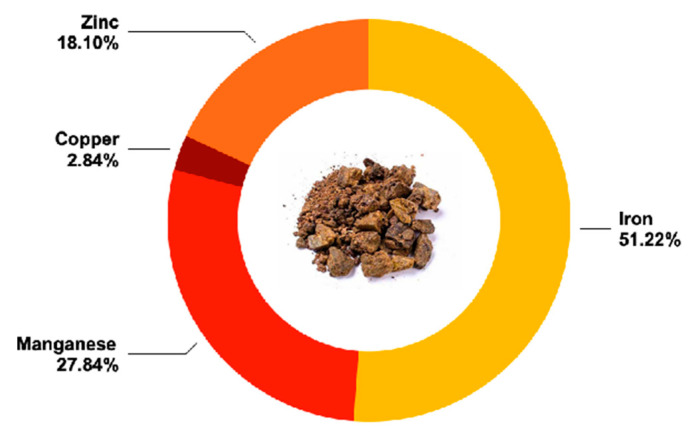

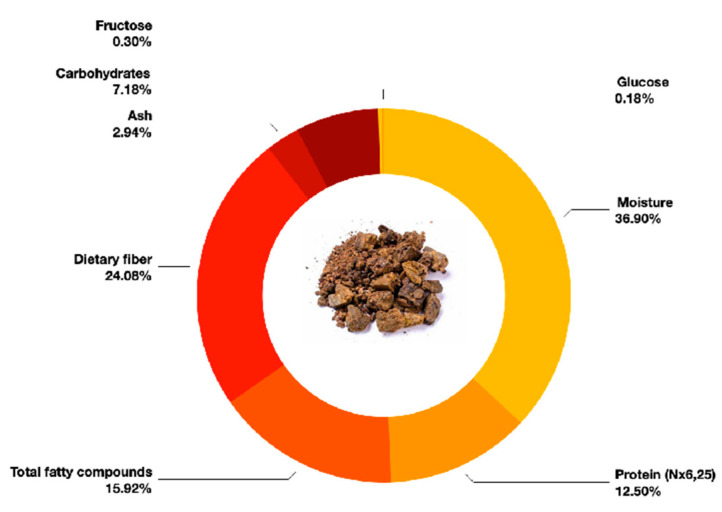
Nutrient and mineral profile of Brazilian propolis residue. This figure presents the nutrient and mineral composition of propolis residue, showcasing high levels of essential elements such as phosphorus (2520 mg/kg), potassium (9800 mg/kg), magnesium (1210 mg/kg), and calcium (2100 mg/kg). The graph emphasizes the propolis residue’s value as a nutritional supplement and its potential in health applications.

Table 4 and Figure 2 detailed the comprehensive nutrient profile of propolis residue. This analysis revealed a significant moisture content (37.08%), underscoring the residue’s potential utility in applications requiring high moisture retention, such as cosmetic formulations or as a natural moisturizing agent in skincare products. This is relevant considering the growing demand for natural and sustainable ingredients in the cosmetic industry [61]. Also, the protein content was high (12.56%), indicating propolis residue as a promising protein source, comparable to or even surpassing that of some plant-based proteins, which typically ranges from 1% to 25% depending on the source [62,63]. This protein content resulted as notable when compared to other unconventional protein sources, such as insect protein, which can vary widely (5–77%) depending on the species [64]. The total fatty compounds (16%) and dietary fiber (24.2%) contents are important, suggesting the residue’s potential in promoting heart and digestive health [65] and its potential use in functional foods for gut health. In addition, the fatty compounds content of propolis residue resulted lower than other sources like nuts and seeds, and it includes potentially beneficial unsaturated fats. Essential fatty acids such as linoleic acid, α-linolenic acid, eicosapentaenoic acid, and docosahexaenoic acid were identified in propolis samples from Northern India [66]. The dietary fiber content was high, surpassing common sources like oats and barley, which are known for their fiber contributions (10–15%). The carbohydrate and the total sugars content (7.21% and 0.482%, respectively) suggested a moderate energy value, making of the residue a potential ingredient in formulations that control energy intake, suggesting a low glycaemic index, meaning that it may help to moderate blood sugar levels by causing slower and more gradual increases—beneficial for maintaining energy and managing health conditions like diabetes.

#### 3.4.2. Micronutrient Profile

The mineral analysis highlighted a good spectrum of essential minerals within the propolis residue, explained in Table 4 and Figure 2. The mineral profile of propolis residue is particularly rich, with high levels of essential minerals such as phosphorus (2520 mg/kg). Specifically, potassium was notably high at 9800 mg/kg, indicating the propolis residue’s potential in supporting cardiovascular health and muscle function. Furthermore, the richness in magnesium (1210 mg/kg) and calcium (2100 mg/kg) can support its application in bone health and metabolic processes. These values highlight the potential utility of propolis residue in mineral supplementation, as it can provide substantial amounts of essential minerals. For instance, the calcium content (2100 mg/kg) is comparable to many dairy products, which typically contain between 400 and 1200 mg per 100 g (equivalent to 4000–12,000 mg/kg). The magnesium content (1210 mg/kg) and phosphorus content (2520 mg/kg) are also significant when compared to typical cereal contents of 150–350 mg/kg and 250–350 mg/kg, respectively [67,68]. Notably, the potassium content (9800 mg/kg) is much higher than that found in bananas, which contain approximately 358 mg per 100 g (equivalent to 3580 mg/kg) [69].

## 4. Conclusions

The comprehensive characterization of Brazilian propolis residue proposed, for the first time, in this study, has unveiled an important resource rich in bioactive compounds, essential nutrients and different volatile elements, positioning it for sustainable reutilization. This residue is particularly noted for its potent antioxidant capacity, as evidenced by the presence of significant levels of *p*-coumaric and derivatives and chlorogenic and caffeic acids. Nutritional profiling revealed considerable protein and dietary fiber content, suggesting its potential application in nutraceuticals and as a food enhancement, pending validation through bioavailability, safety, and regulatory studies. In addition, this matrix boasts a mineral content with a great amount of potassium, phosphorus, and calcium.

On the other hand, the volatile compound analysis opens prospects for natural flavoring and fragrance uses, due to the abundance of terpenoids. This initial exploration into the valorization of propolis residue aligns with the circular economy principles and signals a shift towards more resource-efficient industry practices. Further investigations are encouraged to refine extraction processes, exploring greener methods, with the aim of delivering safe, high-quality extracts for nutraceutical use, thus transforming this still unknown waste into a valuable commodity. Promising techniques include supercritical fluid extraction (SFE) using CO_2_, subcritical water extraction (SWE), ultrasound-assisted extraction (UAE), and natural deep eutectic solvents (NaDES), which offer enhanced selectivity, reduced solvent usage and lower environmental impact.

## Figures and Tables

**Table 1:** Contents of the bioactive phenolic compounds (mg/kg dry weight) determined by HPLC-MS/MS in propolis residue, including Artepillin-C quantified using HPLC-DAD.

Compounds	Propolis Residue ^2,3^
Gallic acid	7.49 ± 0.89 ^a^
Neochlorogenic acid	207.67 ± 19.32 ^b^
Delphinidin 3-galactoside	n.d. ^1^
Catechin	n.d.
Procyanidin B2	n.d.
Chlorogenic acid	497.93 ± 28.33 ^c^
4-Hydroxybenzoic acid	14.37 ± 1.05 ^a^
Epicatechin	n.d.
Cyanidin-3-glucoside	0.14 ± 0.01^a^
Petunidin-3-glucoside	n.d.
3-Hydroxy benzoic acid	n.d.
Caffeic acid	115.11 ± 6.51 ^a^
Vanillic acid	2.69 ± 0.08 ^a^
Pelargonidin-3-glucoside	n.d.
Pelargonidin-3-rutinoside	n.d.
Malvidin-3-galactoside	n.d.
Syringic acid	n.d.
Procyanidin A2	n.d.
*P*-Coumaric acid	637.80 ± 27.35 ^d^
Ferulic acid	n.d.
Rutin	5.59 ± 0.47 ^a^
Hyperoside	104.16 ± 5.61 ^a^
Isoquercitrin	34.75 ± 2.30 ^a^
Delphindin 3,5 diglucoside	34.56 ± 3.17 ^a^
Phloridzin	n.d.
Naringin	n.d.
Quercitrin	0.82 ± 0.04 ^a^
Myricetin	n.d.
Kaempferol-3-glucoside	5.70 ± 0.32 ^a^
Hesperidin	n.d.
Ellagic acid	23.29 ± 2.40 ^a^
*Trans*-cinnamic acid	17.13 ± 0.80 ^a^
Quercetin	31.21 ± 29.94 ^a^
Phloretin	n.d.
Kaempferol	295.82 ± 11.67 ^e^
Isorhamnetin	5.91 ± 0.39 ^a^
TOTAL	2041.34
Artepillin-C	56.56 ± 1.75 mg/kg

^1^ n.d.: not detected. ^2^ Relative standard deviation (RSD) for all compounds ranged from 2.26 to 15.08%. ^3^ All compounds reported significant differences by ANOVA test (*p* < 0.05). Different letters indicate significant differences in Fisher’s LSD test (*p* < 0.05).

**Table 2:** Total phenolic and flavonoid contents and antioxidant activity determined by UV–Vis spectrophotometric analysis in propolis residue.

	Total Phenolic Content (TPC) mg GAE/kg DW	Total Flavonoid Content (TFC) mg RE/kg DW	Antioxidant Activity (DPPH) mg TE/kg DW
Propolis Residue	16,726.67 ± 36.07 ^a^	6802.96 ± 32.40 ^b^	2921.61 ± 24.04 ^c^

TPC was expressed as gallic acid equivalent per kg dry material (mg GAE/kg DW), TFC was expressed as mg of rutin equivalent per kg dry material (mg RE/kg DW), antioxidant activity (DPPH method) was expressed as mg Trolox equivalent per kg dry material (mg TE/kg DW). Different letters indicate significant differences by ANOVA test (*p* < 0.05).

**Table 3:** Chemical composition (%) of volatile compounds identified in propolis residue.

RT ^1,2^	Compounds	RI	RI lit	%
7.286	Ethyl acetate	881.4193	884	1.21
7.458	Diethyl acetal	890.7367	898	5.03
9.355	Ethyl 2-methylpropanoate	964.9116	960	1.57
9.689	Isobutanal diethyl acetal	977.4727	976	0.24
10.853	α-pinene	1020.207	1020	20.09
11.273	Ethyl butanoate	1035.229	1040	2.28
11.787	Ethyl 2-methylbutanoate	1053.612	1057	0.59
12.232	Ethyl 3-methylbutanoate	1069.528	1072	0.34
13.346	β-pinene	1108.049	1108	6.79
14.647	3-carene	1148.018	1146	6.20
15.08	β-myrcene	1161.321	1160	0.39
15.161	α-phellandrene	1163.81	1164	0.76
15.615	α-Terpinene	1177.757	1176	0.38
16.206	D-limonene	1195.914	1196	7.60
16.514	β-phellandrene	1205.686	1201	0.87
17.623	γ-terpinene	1241.715	1243	0.92
17.739	cis-β-Ocimene	1245.484	1245	1.19
17.88	styrene	1250.065	1250	0.16
18.356	o-cymene	1265.53	1268	6.34
23.147	acetic acid	1434.643	1433	0.28
23.747	α-cubebene	1457.535	1458	0.67
24.479	ylangene	1485.464	1485	1.38
24.727	copaene	1494.926	1493	6.93
25.301	benzaldehyde	1517.978	1520	0.38
25.438	(-)-β-bourbonene	1523.563	1517	1.60
27.442	caryophyllene	1605.558	1604	9.76
27.699	aromandendrene	1616.631	1610	4.64
28.628	alloaromadendrene	1656.657	1662	0.83
29.181	humulene	1680.483	1681	1.39
29.536	γ-muurolene	1695.778	1695	3.99
30.315	α-muurolene	1730.814	1727	2.51
31.189	γ-cadinene	1770.362	1767	2.10
34.567	α-calacorene	1929.507	1936	0.59

^1^ R.T. means retention time. R.I. means retention index. R.I. lit means retention index in literature. ^2^ Relative standard deviation (RSD) for all compounds ranged from 2.7 to 8.25%.

**Table 4:** Nutrient (%) and mineral profile (mg/kg) of propolis residue (per 100 g).

Nutrients		
Moisture	g/100 g	37.08 ± 1.07 ^i^
Protein (Nx6,25)	g/100 g	12.56 ± 0.75 ^m^
Total fatty compounds	g/100 g	16.0 ± 1.3 ^l^
Dietary fiber	g/100 g	24.2 ± 3.9 ^k^
Ash	g/100 g	2.95 ± 0.20 ^p^
Carbohydrates	g/100 g	7.21 ± 4.32 ^n^
Fructose	g/100 g	0.299 ± 0.06
Glucose	g/100 g	0.183 ± 0.04
Sucrose	g/100 g	n.d. ^1^
Maltose	g/100 g	n.d.
Lactose	g/100 g	n.d.
**Total Sugars**	g/100 g	0.482 ± 0.07 ^q^
**Energy value**	kCal/100 g	271 ± 12 ^f^
	kJ/100 g	1122 ± 48 ^e^
**Minerals**		
Calcium	mg/kg	2100 ± 290 ^c^
Iron	mg/kg	92 ± 18 ^g^
Phosphorus	mg/kg	2520 ± 470 ^b^
Magnesium	mg/kg	1210 ± 230 ^d^
Manganese	mg/kg	50 ± 11 ^h^
Potassium	mg/kg	9800 ± 1900 g ^a^
Copper	mg/kg	5.1 ± 1.1
Selenium	mg/kg	n.d.
Sodium	mg/kg	n.d.
Zinc	mg/kg	32.5 ± 6.3 ^j^

^1^ n.d.: not detected. The letters in the table represent a ranking of nutrients based on their average values, from highest to lowest. This classification facilitates the visualization of nutrients with the highest mean concentration.

## Data Availability

The original contributions presented in this study are included in the article/Supplementary Material. Further inquiries can be directed to the corresponding author.

## Supplementary Materials

The following supporting information can be downloaded at https://www.mdpi.com/article/10.3390/plants14131989/s1, Figure S1: Schematic representation of the ethanolic extraction process of raw propolis and generation of industrial residue; Table S1: HPLC-MS/MS acquisition parameters (dynamic-MRM mode) used for the analysis of the marker compounds.
