# Supplementary material for: Valorizing Brazilian Propolis Residue: Comprehensive Characterization for Sustainable Reutilization Strategies
Source: Plants (Basel). 2025 Jun 29;14(13):1989. doi: 10.3390/plants14131989 (PMC12252441; doi:10.3390/plants14131989)

**Table S1.** HPLC-MS/MS acquisition parameters (dynamic-MRM mode) used for the analysis of the marker compounds.

| No. | Compounds | Precursor ion, *m/z* | Product ion, *m/z* | Fragm-entor, V | Collision energy, V | Polarity | Retention time (Rt, min) | Delta retention time (ΔRt) |
|---|---|---|---|---|---|---|---|---|
| 1 | Gallic acid | 169 | 125.2* | 97 | 12 | Negative | 6.96 | 2 |
| 2 | Neochlorogenic acid | 353 | 191.2*, 179 | 82 | 12, 12 | Negative | 9.52 | 2 |
| 3 | Delphindin-3-galactoside | 465.01 | 303* | 121 | 20 | Positive | 11.36 | 2 |
| 4 | (+)-Catechin | 289 | 245.2*,109.2 | 131 | 8, 20 | Negative | 11.44 | 2 |
| 5 | Procyanidin B2 | 576.99 | 576.99*, 321.2 | 160 | 0, 32 | Negative | 12.41 | 2 |
| 6 | Chlorogenic acid | 353 | 191.2*, 127.5 | 82 | 12, 20 | Negative | 12.42 | 2 |
| 7 | *p*-Hydroxybenzoic acid | 137 | 93.2* | 92 | 16 | Negative | 12.86 | 2 |
| 8 | (-)-Epicatechin | 289 | 245.1*, 109.1 | 126 | 8, 20 | Negative | 13.03 | 2 |
| 9 | Cyanidin-3-glucoside | 449 | 287.3*, 255.6 | 121 | 20, 20 | Positive | 13.14 | 2 |
| 10 | Petunidin-3-glucoside | 479.01 | 317*, 302 | 121 | 20, 44 | Positive | 13.26 | 2 |
| 11 | 3-Hydroxybenzoic acid | 137 | 93.2* | 88 | 8 | Negative | 13.59 | 2 |
| 12 | Caffeic acid | 179 | 135.2*, 134.1 | 92 | 12, 24 | Negative | 13.65 | 2 |
| 13 | Vanillic acid | 167 | 152.4*, 108.1 | 88 | 12, 20 | Negative | 14.32 | 2 |
| 14 | Pelargonidin-3-glucoside | 433.01 | 271*, 121 | 116 | 24, 50 | Positive | 14.52 | 2 |
| 15 | Pelagonidin-3-rutinoside | 579.01 | 271* | 145 | 32 | Positive | 14.56 | 2 |
| 16 | Malvidin-3-galactoside | 493.01 | 331*, 315.1 | 121 | 20, 50 | Positive | 14.64 | 2 |
| 17 | Syringic acid | 196.9 | 182.2*, 121.2 | 93 | 8, 12 | Negative | 15.28 | 2 |
| 18 | Procyanidin A2 | 575 | 575*, 285 | 170 | 0, 20 | Negative | 16.18 | 2 |
| 19 | *p*-Coumaric acid | 163 | 119.2*, 93.2 | 83 | 12, 36 | Negative | 16.70 | 2 |
| 20 | Ferulic acid | 193 | 134.2*, 131.6 | 83 | 12, 8 | Negative | 17.10 | 2 |
| 21 | 3,5-Dicaffeoylquinic acid | 514.9 | 353.1*, 191 | 117 | 8, 28 | Negative | 17.61 | 2 |
| 22 | Rutin | 609 | 300.2*, 271.2 | 170 | 32, 50 | Negative | 17.73 | 2 |
| 23 | Hyperoside | 465.01 | 303*, 61.1 | 97 | 8, 50 | Positive | 18.33 | 2 |
| 24 | Isoquercitrin | 463 | 271.2*, 300.2 | 155 | 44, 24 | Negative | 18.36 | 2 |
| 25 | Delphindin-3,5-diglucoside | 462.9 | 300.1* | 165 | 24 | Negative | 18.38 | 2 |
| 26 | Phloridzin | 435.39 | 273*, 167 | 155 | 8, 28 | Negative | 18.83 | 2 |
| 27 | Quercitrin | 446.99 | 300.2*, 301.2 | 160 | 24, 16 | Negative | 19.61 | 2 |
| 28 | Myricetin | 316.99 | 179.1*, 182 | 150 | 16, 24 | Negative | 19.61 | 2 |
| 29 | Naringin | 578.99 | 271.3*, 151.3 | 170 | 32, 44 | Negative | 19.62 | 2 |
| 30 | Kaempferol-3-glucoside | 447 | 284.2*, 255.2 | 170 | 24, 40 | Negative | 19.77 | 2 |
| 31 | Hesperidin | 611.01 | 303*, 334.8 | 112 | 20, 12 | Positive | 20.19 | 2 |
| 32 | Ellagic acid | 301 | 301*, 229 | 170 | 0, 24 | Negative | 21.41 | 2 |
| 33 | Quercetin | 300.99 | 151.2*, 179.2 | 145 | 16, 12 | Negative | 21.87 | 2 |
| 34 | Phloretin | 272.99 | 167*, 123 | 116 | 8, 20 | Negative | 22.30 | 2 |
| 35 | Kaempferol | 287.01 | 153*, 69.1 | 60 | 36, 50 | Positive | 23.84 | 2 |
| 36 | Isorhamnetin | 314.99 | 300.2*, 196.1 | 145 | 16, 4 | Negative | 24.57 | 2 |

* These product ions were used for quantification.

Figure S1. Schematic representation of the ethanolic extraction process of raw propolis and generation of industrial residue.

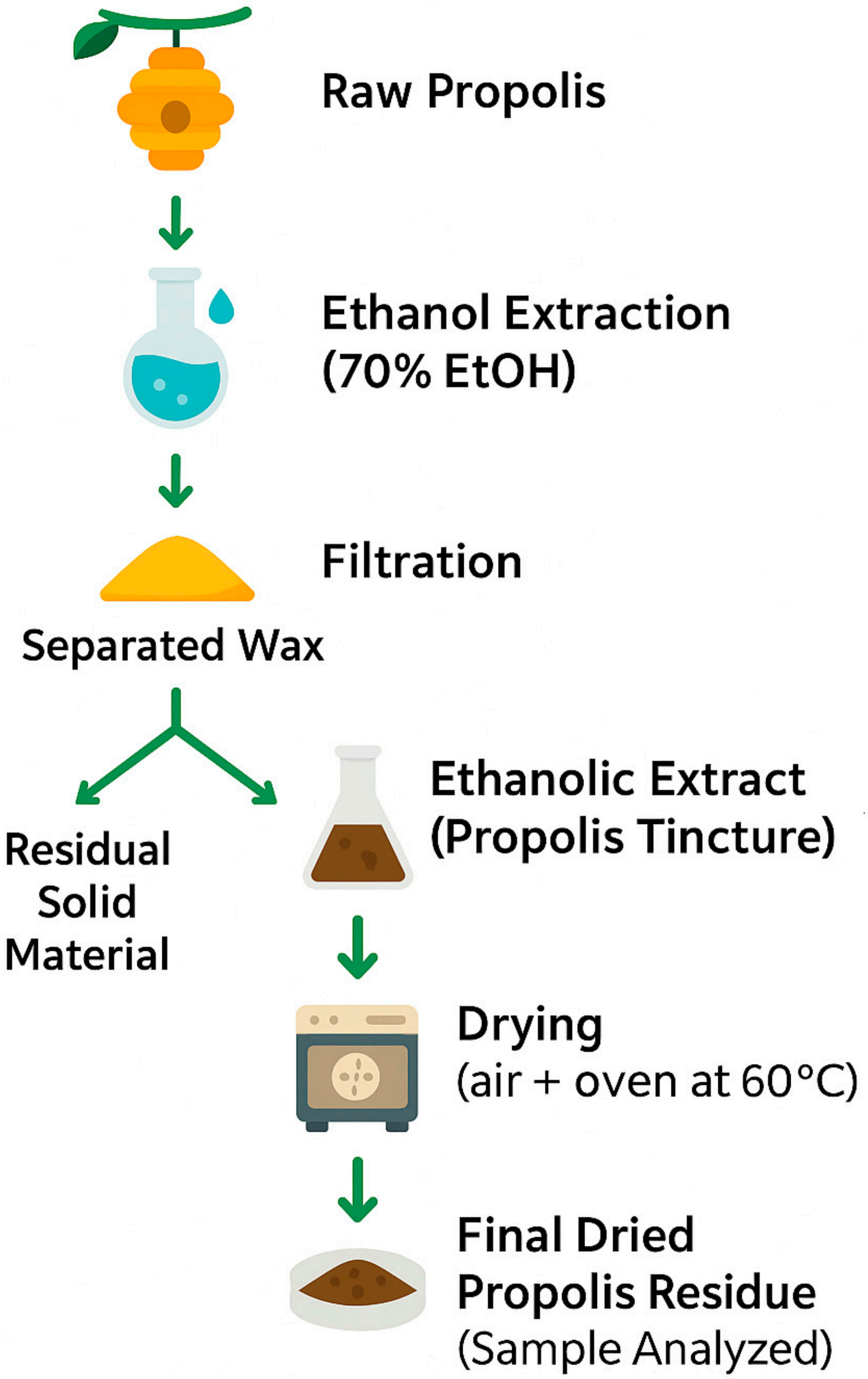

Supplement: Supplementary file 1 [file plants-14-01989-s001.zip › plants-3709324-supplementary.pdf]
